# Postherpetic Neuralgia and Trigeminal Neuralgia

**DOI:** 10.1155/2017/1681765

**Published:** 2017-12-05

**Authors:** L. Feller, R. A. G. Khammissa, J. Fourie, M. Bouckaert, J. Lemmer

**Affiliations:** ^1^Department of Periodontology and Oral Medicine, Sefako Makgatho Health Sciences University, Pretoria, South Africa; ^2^Department of Maxillofacial and Oral Surgery, Sefako Makgatho University, Pretoria, South Africa

## Abstract

Postherpetic neuralgia (PHN) is an unpredictable complication of varicella zoster virus- (VZV-) induced herpes zoster (HZ) which often occurs in elderly and immunocompromised persons and which can induce psychosocial dysfunction and can negatively impact on quality of life. Preventive options for PHN include vaccination of high-risk persons against HZ, early use of antiviral agents, and robust management of pain during the early stage of acute herpes zoster. If it does occur, PHN may persist for months or even years after resolution of the HZ mucocutaneous eruptions, and treatment is often only partially effective. Classical trigeminal neuralgia is a severe orofacial neuropathic pain condition characterized by unilateral, brief but recurrent, lancinating paroxysmal pain confined to the distribution of one or more of the branches of the trigeminal nerve. It may be idiopathic or causally associated with vascular compression of the trigeminal nerve root. The anticonvulsive agents, carbamazepine or oxcarbazepine, constitute the first-line treatment. Microvascular decompression or ablative procedures should be considered when pharmacotherapy is ineffective or intolerable. The aim of this short review is briefly to discuss the etiopathogenesis, clinical features, and treatment of PHN and classical trigeminal neuralgia.

## 1. Postherpetic Neuralgia

### 1.1. Introduction

Herpes zoster (HZ) is an acute, localized self-limiting infection caused by the varicella zoster virus (VZV), a neurotropic alpha-herpes virus, most frequently affecting elderly or immunocompromised persons. Reactivation of latent VZ acquired during previous episodes of VZV infection (chickenpox) and which have persisted in a latent form within the dorsal root ganglia neural cells results in viral replication followed by spread of the virus down the sensory nerve to the skin/mucosa. The reactivated VZV can then cause unilateral vesicular eruptions with acute, stabbing radiating pain, confined to a dermatome [1–6]. Typically the cutaneous HZ starts as localized prodromal pain, and then discrete patches of erythema appear where vesicles develop, rupture, and become crusted all within seven to ten days, though the lesions may take a month or more to heal [1, 7]. Oral mucosal eruptions of HZ have a similar clinical course to that of the cutaneous lesions but owing to the wet environment do not crust.

The mucocutaneous damage is thought to be the consequence of the direct cytopathic effect of VZV on epithelial cells [3, 4, 8, 9]. The pain of the acute phase of HZ is caused by VZV-induced cytopathic damage to nerve cells in the sensory ganglia and in the peripheral sensory nerves during the descent of the reactivated VZV. The intense inflammatory reaction to the descending VZV affects both the neural and the mucocutaneous tissues so that the pain of the neuritis is exaggerated by inflammatory pain [1, 9].

The vesicular mucocutaneous eruptions of HZ are usually preceded by two or three days of localized pain, and the acute neuritis that accompanies the eruptions may sometimes leave the legacy of postherpetic neuralgia (PHN). PHN is thus a complication of HZ and is characterized by symptoms of neuropathic pain including intense burning sensation, allodynia, and/or hyperalgesia that may continue long after resolution of the mucocutaneous eruption [1, 2]. PHN is a debilitating condition with an impact on both physical and emotional functions, reducing the quality of life of PHN sufferers [6].

About 10 to 15% of persons with HZ will develop PHN. This is uncommon under the age of 40 years, but in HZ-affected persons older than 60 years, it occurs in more than 50% [10]. The more intense the mucocutaneous eruptions of HZ and the acute herpetic neuritis pain during the acute phase of HZ, the greater the frequency and intensity of the PHN [2].

Available evidence-based information about the efficacy of different treatment modalities for PHN is sparse [6], so treatment is largely based on expert opinion. Fortunately PHN is eventually self-limiting.

### 1.2. Clinical Features of Postherpetic Neuralgia

PHN is that pain which in some cases persists in the dermatome affected by mucocutaneous HZ after resolution of the vesiculopapular rash. In most cases, PHN resolves spontaneously over weeks or months from the resolution of the rash, but sometimes, the pain may persist longer. It has been reported that rarely PHN may have its onset months or even years after the initial episode of HZ has resolved [6].

Postherpetic neuralgic pain is characterized by allodynia and hyperalgesia in response to nonnoxious mechanical and thermal stimuli and by spontaneous pain variably described as burning, sharp, shooting, or electric shock-like [4]. Within the affected dermatomes there may be areas of diminution of awareness of sensations of vibration, pinprick, or heat [6]. Fatigue, anorexia, weight loss, insomnia, reduced physical activity, depression, anxiety, and a decrease in social contacts are commonly associated with PHN [6].

### 1.3. Pathogenesis of Postherpetic Neuralgia

Reactivation and subsequent replication of latent VZ viruses (VZVs) in neural cells of the dorsal root ganglia induce both direct cytopathic damage to central and peripheral neurons, and VZV-mediated inflammatory tissue damage will exaggerate the neuralgic pain [2, 6]. The VZV-induced ganglionitis and neuritis during the acute stage of the HZ stimulate a robust local sympathetic reaction which causes vasoconstriction with consequent ischaemic nerve damage and pain, thus contributing to the intensity of the acute neuritic pain [11].

These biopathological events during the acute stage of HZ can in time cause both peripheral and central sensitisation characterized by downregulation of central pain inhibitory pathways, alterations in expression of genes encoding neuropeptides, and expansion of receptive fields. This results in hyperexcitability of dorsal horn neurons with the capacity to fire spontaneously. These sensory functional alterations within the neurons account for the allodynia, hyperalgesia, burning, and electric shock-like sensations characteristic of postherpetic neuralgic pain [2, 6, 11]. Furthermore, the VZV-induced neuronal damage can also lead to transient or permanent diminution in the sensing of certain stimuli [5]. However, the relationship between the nature of the VZV-induced sensory dysregulation and the variable characteristics of the pain is not clear [3].

### 1.4. Treatment

There is no effective and predictable treatment for PHN [6, 7]. Vaccination of persons with a history of HZ, the use of appropriate antiviral agents within the first 72 hours of the onset of the HZ rash, and adequate pain control during the acute stage of the neuritis are the best preventive treatment options for PHN [6].

A single dose of live attenuated VZV vaccine will boost virus-specific cell-mediated immunity, and in those who develop HZ despite the vaccination, it will reduce the incidence of PHN [12]. Nonsteroidal anti-inflammatory agents (NSAIDs) and opioids, alone or in combination, have been used to control the acute pain of HZ, but with equivocal reports of beneficial effects [6]. Taken orally, the nucleoside analogues acyclovir, famciclovir, and valacyclovir which inhibit replication of VZV, can reduce the severity and duration of the acute neuritic pain, thus reducing the risk of PHN [12]. It has also been demonstrated that blockade of the sympathetic ganglia, together with NSAIDs and antiviral agents early in the course of acute HZ, decreases the intensity of the acute neuritic pain and consequently reduces the frequency of PHN [11].

In established PHN, a multimodal approach is usually necessary for a good clinical outcome. This includes a combination of topical and systemic medications, relaxation, psychological intervention, and social support. Topical medications used include anaesthetic agents, capsaicin, and various anti-inflammatory preparations [3]; systemic medications include opioids, anticonvulsants (calcium channel blockers, e.g., gabapentinoids, and sodium channel blockers, e.g., phenytoin), antidepressants (noradrenalin and serotonin reuptake inhibitors, e.g., duloxetine and venlafaxine), and tricyclic antidepressants (e.g., amitriptyline, nortriptyline or desipramine) [13] (detailed information for the therapeutic management of PHN can be found in Johnson and Rice 2014 [13]; Hempenstall et al., 2005 [14]; Dworkin et al., 2007 [15]).

It appears that drug-combination therapy is more effective and better tolerated than the use of a single drug in reducing the intensity of PHN pain [6, 16], and it has been shown that psychological, social, and spiritual support all have a positive effect on the overall quality of life of PHN sufferers [6]. Peripheral or sympathetic nerve blocks, cryotherapy, acupuncture, biofeedback, and transcutaneous electrical stimulation are other options that may be useful and may complement the conventional treatment of PHN [7].

## 2. Trigeminal Neuralgia

### 2.1. Introduction

Trigeminal neuralgia (tic douloureux) is an orofacial pain disorder characterized by unilateral, recurrent paroxysms of lancinating pain confined to the somatosensory distribution of the trigeminal nerve. The second and third divisions of the trigeminal nerve are more frequently affected than the first division. Typically, an attack of pain is elicited by innocuous tactile stimuli to trigger points somewhere in the distribution of the trigeminal nerve [17–19]. Activities such as chewing, talking, smiling, grimacing, washing, shaving, and brushing teeth as well as light touch can set off an attack of trigeminal neuralgia [19, 20]. About 4% of persons with trigeminal neuralgia have the condition bilaterally: attacks of pain only rarely affect both sides [21–23].

At the onset of a paroxysm of trigeminal neuralgia, the pain reaches peak intensity immediately and lasts a few seconds to one or two minutes, and the lancinating pain is sometimes accompanied by involuntary spasms of the facial muscles (tic douloureux) [19, 24]. Occasionally mild autonomic symptoms such as lacrimation and/or redness of the eye may occur concurrently with a trigeminal neuralgic attack [25]. Following an episode of pain there is a refractory phase of several minutes during which there will not be another paroxysm of pain even if the triggering stimulus recurs [19]. Typically, trigeminal neuralgia does not interfere with sleep. It affects females more often than males, its prevalence increases with age, and the annual incidence is estimated to be between 4 and 13 per 100,000 [19, 24]. Curiously, the right side of the face/mouth is more commonly affected [21–23].

The clinical course of trigeminal neuralgia is characterized by a progressive increase in frequency, duration, and severity of attacks, gradual diminution of response to medication, and sometimes the development of constant dull pain between attacks. However, there may be spontaneous remissions of six months or more [20, 22].

Classical (typical) trigeminal neuralgia is either idiopathic or is associated with vascular compression of the root of the trigeminal nerve close to its point of entry into the pons (the “root entry zone”), by an aberrant arterial or venous loop [20, 26, 28] (Box 1). At the level of the trigeminal “root entry zone,” the myelin is of the “central type” produced by oligodendrocytes, probably less resistant to mechanical stimuli than peripheral myelin produced by Schwann cells [22]. Furthermore, following injury, the extent and quality of oligodendrocytic-induced remyelination are suboptimal [29], thus making the trigeminal root entry zone vulnerable to the development of aberrant neural activity.

**Figure 1:**
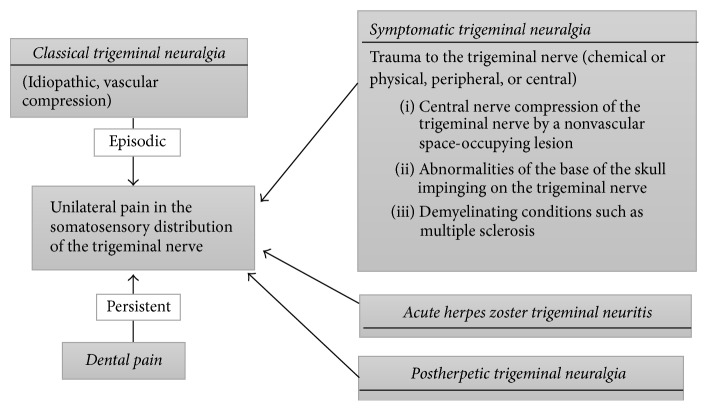
Orofacial neuralgic pain conditions that can occur in the somatosensory distribution of the trigeminal nerve. Careful examination, a thorough medical and dental history, and neuroimaging of the head with magnetic resonance imaging (MRI) are necessary for the diagnosis of classical and symptomatic trigeminal neuralgia [18, 24].

Trigeminal neuralgia can also be symptomatic if it is associated with some other cause such as multiple sclerosis or nonvascular space-occupying lesions in the brain [30, 31] (Figure 1). Symptomatic trigeminal neuralgia is associated with an increased risk of bilateral neuralgic pain and is characterized by dull continuous pain between paroxysms, neurological abnormalities in the trigeminal nerve distribution, and younger age, usually under 40 [18, 19, 22, 24, 32]. In persons with multiple sclerosis, the onset of trigeminal neuralgia occurs at an earlier age, with an increased prevalence ranging from 3.8% to 9.7% and with increased incidence of bilateral manifestation [33, 34]. Differential diagnosis of trigeminal neuralgia is shown in Figure 1. As classical from symptomatic trigeminal neuralgia is not always straightforward, neuroimaging with brain MRI may facilitate the identification of structural brain lesions, demyelinating lesions, and vascular compression at the trigeminal nerve root entry zone, thus allowing for an accurate diagnosis [19].

### 2.2. Pathophysiology of Classical Trigeminal Neuralgic Pain

Classical trigeminal neuralgia can be either idiopathic or caused by vascular compression of the proximal portion of the trigeminal root at the level of the brain stem. The compression causes some ischaemic and mechanical damage with microstructural changes to the trigeminal neurons and with the appearance of focal zones of demyelination [5].

Physiologically, after a sharp response to a brief stimulus, the membranes of sensory nerves rapidly return to a resting state; where patches of demyelination have exposed neuronal membranes, where there are sites of structural anomalies or aberrantly myelinated axonal clusters, the threshold of excitability may not only be lowered but these sites may also act as autonomous pacemakers with the capacity to generate an attack or barrage of impulses in response to brief stimuli [23]. A prolonged after-discharge may generate an electric shock-like pain sensation that outlasts the triggering stimulus by one or two minutes [17]. Eventually, polarization sets in within the abnormally hyperactivated nerve fibres with a rapid fall in excitability and suppression of the firing. There is then a refractory period of two or three minutes during which another burst of neuronal activity cannot be triggered, probably because of a delay in the restoration of ionic gradients after the burst of discharges. These cycles of neuronal hyperactivity and silence may explain the paroxysmal nature of trigeminal neuralgic pain [17, 20, 23].

As a minority of cases of classical trigeminal neuralgia are not caused by vascular compression and appear to be idiopathic, it is possible that disordered physiological, anatomical, genetic, toxic, or degenerative factors may precipitate the generation of abnormal discharges by the Gasserian ganglion, the trigeminal nerve root, or the trigeminal nucleus, causing the neuralgic pain [20, 35]. Altered functional activity of sodium channels along trigeminal nociceptive axons, as in the case of diabetic peripheral neuropathy [36], alterations in the physiological process of the maintenance of the integrity of myelin [28], and microstructural changes in the trigeminal root in the absence of any vascular pressure [28] may lead to the generation of abnormal discharges, playing a role in idiopathic classical trigeminal neuralgic pain.

In zones of trigeminal nerve compression and demyelination, the high-frequency discharge of impulses in the affected neurons may cross-excite neighbouring electrically silent axons, with impulse amplification and the generation of an intense, explosive discharge causing sudden lancinating pain. Furthermore, in zones of trigeminal nerve demyelination, extra-synaptic transmission of electrical impulses from A*β* touch sensation conducting fibres to pain conducting C-fibres may occur, explaining how tactile stimulation of trigger points in persons with trigeminal neuralgia may generate trigeminal neuralgic pain [23]. In this context, it is possible that the mere pulsation of blood vessels in contact with demyelinated or structurally malformed trigeminal axons may initiate aberrant electrical impulses exacerbating the pain [20].

In those cases of trigeminal neuralgia not associated with vascular compression, genetic alterations causing dysfunctional activity of Na_v_ 1.9 channels may mediate the generation of persistent sodium currents at subthreshold voltage, promoting depolarisation and reducing the threshold of the current required to trigger an action potential, resulting in a state of hyperexcitability of sensory neurons [35].

Classical trigeminal neuralgia may be either idiopathic or induced by vascular compression of the trigeminal root. Demyelination probably plays an essential role in the hyperexcitability and abnormally high-frequency repetitive neuronal firing in trigeminal neuralgia, but demyelination per se is probably not sufficient to cause trigeminal neuralgia [22].

It appears that the onset of classical trigeminal neuralgia without evident vascular compression of the root of the trigeminal nerve occurs at a significantly earlier age than does idiopathic trigeminal neuralgia (42 and 51 years, resp.), and regardless of treatment modality, recurrence is more frequent for those cases of trigeminal neuralgia that were not originally caused by vascular compression than for those that were [37].

### 2.3. Treatment

In the treatment of classical trigeminal neuralgia, the anticonvulsants carbamazepine and oxcarbazepine are equally effective and are the drugs of choice [27, 31, 32]. These drugs inhibit the function of voltage-sensitive sodium channels of hyperexcited neuronal membranes, thus raising the threshold of excitability [31, 32], inhibiting the high-frequency repetitive neuronal firing characteristic of trigeminal neuralgia. Baclofen, lamotrigine, gabapentin, phenytoin, and clonazepam are other agents with neural membrane-stabilising properties that may likewise relieve the pain [27]. However, when pharmacotherapy is ineffective, or not well tolerated, interventional microvascular decompression or ablative procedures should be considered.

Microvascular decompression for classical trigeminal neuralgia is a major surgical procedure in which the compressing blood vessels are separated from the trigeminal nerve root in the posterior fossa. Alternatively, sensory fibres of the trigeminal nerve can be ablated, either peripherally or centrally, though pain relief is then at the cost of varying degrees of sensory loss [27]. About 90% of patients experience pain relief soon after microvascular decompression, but relief of pain declines to about 73% after 5 years [18].

Another surgical modality in the treatment of idiopathic classical trigeminal neuralgia is the “nerve combing” method. This involves the longitudinal splitting of the trigeminal root into several fascicles from the entrance to the pons. The speculative biological rationale for pain relief achieved by “nerve combing” is the assumption that the surgical trauma to the trigeminal root somehow affects the dysregulated neural circuits in the brainstem, abolishing their abnormal activity [38–40].

### 2.4. Comments

(i) Contact of blood vessels with the root of the trigeminal nerve is normal, inevitably with some hydrostatic pressure on the nerve, but only if there is vascular pressure on the nerve sufficient to cause neuronal damage, can this become an aetiological factor. In classical trigeminal neuralgia, neuronal microstructural alterations including demyelination can occur independently of any vascular pressure, and it is not clear whether or not there is any correlation between the degree of severity of the neuronal microstructural damage caused by the vascular compression and the idiopathic microstructural alterations unrelated to the vascular compression on the one hand and the severity and duration of the trigeminal neuralgic pain on the other hand [35, 37, 41].

(ii) One of the criteria for the diagnosis of classical trigeminal neuralgia is an absence of any common sensory abnormality in the distribution of the trigeminal nerve. However, in classical trigeminal neuralgia, sometimes routine examination reveals unrelated sensory neurological deviations in the distribution of the trigeminal nerve [41]. It can therefore be argued that if there are no discoverable secondary causes for the trigeminal neuralgia and if all other criteria for classical trigeminal neuralgia are fulfilled, then a diagnosis of classical trigeminal neuralgia can be justified [41].

## 3. Conclusion

Both postherpetic neuralgia and trigeminal neuralgia are painful, debilitating conditions which are often treatment-resistant, causing psychosocial dysfunction and reduced quality of life. A better understanding of the pathogenesis of these incapacitating chronic pain conditions is needed for the development of more effective treatment strategies.

## Figures and Tables

**Box 1:**
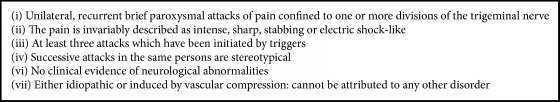
Diagnostic criteria for classical trigeminal neuralgia. Adapted from [18, 22–24, 26, 27].
